# Medical-Grade Poly(Lactic Acid)/Hydroxyapatite Composite Films: Thermal and In Vitro Degradation Properties

**DOI:** 10.3390/polym15061512

**Published:** 2023-03-18

**Authors:** Leonard Bauer, Anamarija Rogina, Marica Ivanković, Hrvoje Ivanković

**Affiliations:** Faculty of Chemical Engineering and Technology, University of Zagreb, Trg Marka Marulića 19, HR-10001 Zagreb, Croatia

**Keywords:** poly(lactic acid), hydroxyapatite, in vitro degradation, microstructure

## Abstract

Production of biocompatible composite scaffolds shifts towards additive manufacturing where thermoplastic biodegradable polymers such as poly(lactic acid) (PLA) are used as matrices. Differences between industrial- and medical-grade polymers are often overlooked although they may affect properties and degradation behaviour as significantly as the filler addition. In the present research, composite films based on medical-grade PLA and biogenic hydroxyapatite (HAp) with 0, 10, and 20 wt.% of HAp were prepared by solvent casting technique. The degradation of composites incubated in phosphate-buffered saline solution (PBS) at 37 °C after 10 weeks showed that the higher HAp content slowed down the hydrolytic PLA degradation and improved its thermal stability. Morphological nonuniformity after degradation was indicated by the different glass transition temperatures (*T*_g_) throughout the film. The *T*_g_ of the inner part of the sample decreased significantly faster compared with the outer part. The decrease was observed prior to the weight loss of composite samples.

## 1. Introduction

Tissue engineering represents an alternative approach for treating bone defects where biodegradable and biocompatible scaffolds act as temporary supports for cell attachment, proliferation and differentiation leading to bone tissue regeneration. Additive manufacturing techniques such as fused deposition modelling (FDM) or fused filament fabrication (FFF) emerged as suitable methods for personalized and integrated bone tissue engineering [1].

As one of the most commonly used biodegradable polymers for bone tissue engineering, poly(lactic acid) (PLA) is considered a thermoplastic polymer well-suited for additive manufacturing of bone scaffolds due to its low melting temperature for melt extrusion [2]. PLA shows desirable properties for tissue-engineering applications, including nontoxicity, biocompatibility, and nontoxic in vitro biodegradation [3]; however, it is unable to form a direct bond with bone tissue in vivo. The addition of bioactive ceramics or bioactive glass is one way to enhance the osteogenic potential of PLA-based scaffolds [4,5,6]. Synthetic calcium phosphates, in particular hydroxyapatite (HAp), are the most commonly used bioactive ceramics in dentistry, orthopaedics, and bone tissue engineering. The fabrication of PLA-HAp filaments used in additive manufacturing is usually done by extrusion of raw materials or composite pellets previously obtained by solvent casting.

The ideal bone scaffold should degrade at a controlled rate in vivo leaving space for new bone tissue growth [7]. PLA is classified as a slow-degrading polymer in vitro and in vivo. The difference in degradation kinetics of PLA-based materials is caused by molecular weight, crystallinity, sample dimensions, microstructure, porosity, filler addition, and purity of raw components [8]. The degradation of PLA is usually described as a two-stage process involving a decrease in molecular weight and the onset of weight loss. The first stage usually occurs at the beginning of degradation, which can be attributed to the random hydrolytic ester cleavage, until reaching the critical molecular weight. At this point, the second stage of degradation is initiated and characterized by the onset of weight loss [3]. Due to slow PLA degradation, in vitro degradation tests of PLA-based composites are performed under physiological conditions for a few months [9], while in vitro degradation experiments at higher temperatures (e.g., 70 °C) could give an insight into degradation kinetics during a few weeks of incubation [10]. Both stages of the PLA degradation mechanism could be accelerated by the incorporation of inorganic fillers of different shapes and sizes. Previous works on the biodegradation of PLA-HAp composites [10,11] indicated accelerated degradation of materials with a higher content of HAp nano- or microparticles. The hydrophobic nature of PLA is responsible for its longer stability under physiological conditions, while the addition of hydrophilic HAp particles enhances medium absorption leading to faster autocatalytic hydrolysis of the polymer. Blending PLA with bioactive ceramics is an easy way to adjust the degradation rate of potential bone scaffolds, which is one of the predominant scaffold properties during tissue regeneration.

The majority of research on PLA-based materials used as potential bone scaffolds was conducted with technical-grade PLA which is a cost-effective feedstock material for 3D-printing applications [12]. Such PLA is characterized as a semi-crystalline polymer with longer degradation under physiological conditions, containing different impurities and additives for easier additive manufacturing [13]. In clinical practices, materials with a strictly defined composition and high purity are required [14]. More importantly, the compositional difference between technical-grade and medical-grade biodegradable polymers leads to different material behaviour in vitro and in vivo, in terms of bioresorption and biodegradation [15,16]. However, such polymers are too expensive to be used in materials science, which has resulted in a few papers on medical-grade PLA-based materials [15]. Here, we report on the in vitro degradation of PLA-HAp composites based on medical-grade PLA and hydroxyapatite synthesized from environmentally friendly biogenic waste materials that are available in large quantities in nature.

In this study, the degradation behaviour of medical-grade poly(lactic acid) modified with hydroxyapatite was examined after a prolonged incubation period in phosphate-buffered saline solution at 37 °C. PLA-HAp composite films with different content of hydroxyapatite (0–20 wt.%) were prepared in order to investigate the influence of the inorganic phase on the degradation properties of medical-grade PLA. The compositional and morphological changes and thermal properties of PLA-HAp composites were investigated during 10 weeks of incubation.

## 2. Materials and Methods

### 2.1. Preparation of Hydroxyapatite

Hydroxyapatite powder was prepared via hydrothermal transformation of cuttlefish bone (*Sepia officinalis* L., Adriatic Sea) which was used as a source of calcium ions [17]. Small samples of cuttlefish bone were cleaned with sodium hypochlorite solution (NaClO, 13% active chlorine; Gram-Mol, Zagreb, Croatia) for 48 h at room temperature and then extensively washed in distilled water. A specific amount of cleaned bone samples was transferred into autoclave reactors with an appropriate volume of 0.6 mol dm^−3^ solution of ammonium dihydrogen phosphate (NH_4_H_2_PO_4_, 99%, Scharlau, Barcelona, Spain), respecting the calcium and phosphorus (Ca/P) molar ratio of 1.67. The reaction was carried out at a temperature of 200 °C for 48 h under self-generated pressure. After 48 h, the obtained samples were washed with hot distilled water and dried at a temperature of 105 °C. Finally, dried samples were milled and sieved to obtain a particle size of 90–125 µm.

### 2.2. Preparation of Poly(Lactic Acid)/Hydroxyapatite Composite Films

Medical-grade poly(lactic acid) (M_W_ ≈ 60 000, FP158009, Biosynth, Compton, UK) was dissolved in ethyl acetate (HPLC grade, Fischer Chemical, Leicester, UK) resulting in a 10 *w*/*v*% polymer solution. After the PLA was completely dissolved, hydroxyapatite was added to the polymer solution. Ultrasonic probe Sonoplus HD4200 (Bandelin, Berlin, Germany) with a UW200 converter and TT 213 sonotrode at 40% amplitude was used 3 times for 2 min to assist in dispersing HAp particles within the solution.

Composite PLA-HAp samples were prepared by varying the HAp weight content: 100/0 (PLA), 90/10 (PLA-10-HAp), and 80/20 (PLA-20-HAp). Polymer solutions were concentrated at 50 °C with mild magnetic stirring until 2/3 of the ethyl acetate volume evaporated. Thus, prepared dense polymer solutions were poured into concave silicone moulds to obtain films with reproducible shape and weight. The prepared films were dried at 50 °C for 24 h to remove the solvent. The solvent-casted films with a maximum thickness of 0.5 mm, diameter of ~20 mm and weight of 105 ± 15 mg were obtained.

### 2.3. In Vitro Degradation Experiment

The degradation behaviour of PLA-HAp composite films was investigated in phosphate-buffered saline solution (PBS, pH 7.4) according to ISO 13781:2017 (E) standard. Weighed samples were immersed in 10 mL of PBS supplemented with 0.2 mg mL^−1^ of biocide (NaN_3_, 99+% AnalR NORMAPUR, VWR Chemicals, Leuven, Belgium) for 10 weeks at 37 °C. Triplicates of PLA, PLA-10-HAp and PLA-20-HAp composites were prepared for incubation periods of 2, 4, 5, 6, 7, 8, 9, and 10 weeks.

The incubation medium was replaced by fresh PBS solution twice a week. At a specific incubation time, samples were carefully collected from the degradation medium, washed with distilled water, and dried at 50 °C until constant mass was obtained. The degradation degree was estimated as a weight loss determined at a specific time point with respect to the initial weight.

### 2.4. Identification and Characterization of Poly(Lactic Acid)/Hydroxyapatite Composite Films

The identification of materials was carried out by X-ray diffraction analysis using an XRD-6000 diffractometer (Shimadzu, Kyoto, Japan) with Cu K_α_ radiation operated at 40 kV and 30 mA, in the range of diffraction angles (2θ) 5–70° at a scan speed of 0.2° s^−1^. The reference cards for HAp, brushite and halite standards (9-432, 9-77, and 5-628, respectively), compiled by the International Centre for Diffraction Data (ICDD, Newton Square, PA; USA) were used for crystal phase identification.

The morphology of materials was examined by scanning electron microscopy (SEM Tescan Vega III Easyprobe, Tescan Orsay Holding, Brno, Czech Republic). An energy-dispersive X-ray (EDX) spectrometer (Bruker B-Quantax, Bruker Nano GmbH, Berlin, Germany) connected to the SEM has been used to determine the elemental composition of scaffolds. Prior to the SEM and EDX analysis, the samples were sputtered with gold and palladium for 60 s.

Thermogravimetric analysis (TGA) was performed on a Netzsch STA 409 (Netzsch Instruments, Selb, Germany) with a constant synthetic airflow of 30 cm^3^ min^−1^ from 40 °C to 1200 °C at a heating rate of 10 °C min^−1^. Differential scanning calorimetry was performed on a DSC 3500 Sirius Netzsch (Netzsch Instruments, Selb, Germany) equipped with a special cooler IC70 Netzsch with a constant nitrogen flow of 50 cm^3^ min^−1^. Two continuous heating—cooling cycles were conducted at a heating rate of 10 °C min^−1^. The first cycle from 20 °C to 220 °C and back to −20 °C was used to erase the sample thermal history, while the inflexion point of the endothermic peak in the second cycle from −20 °C to 220 °C was determined as the glass transition temperature.

## 3. Results and Discussion

### 3.1. Weight Loss

**Figure 1:**
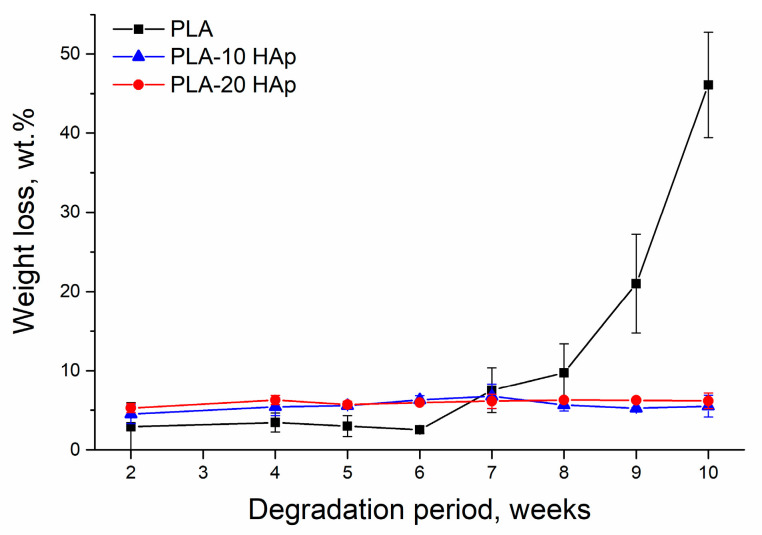
Weight loss of PLA, PLA-10-HAp, and PLA-20-HAp samples during the in vitro degradation process performed in phosphate-buffered saline solution (PBS, pH 7.4) supplemented with a biocide (NaN_3_, concentration of 0.2 mg mL^−1^) under static conditions at 37 °C for 10 weeks.

The weight loss of PLA and PLA-HAp composite films is shown in Figure 1. In the first period of degradation, all samples showed a slight linear weight loss. Until week 6, PLA sample lost 3 wt.%, while both composite samples’ weight loss reached 6 wt.%. After week 7, weight loss for PLA-10-HAp and PLA-20-HAp samples remained close to 6 wt.%, while PLA sample weight loss exponentially increased up to 46 wt.%.

### 3.2. XRD Analysis

**Figure 2:**
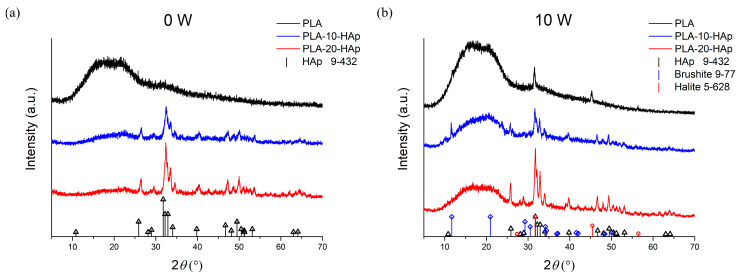
XRD patterns of PLA, PLA-10-HAp and PLA-20-HAp materials (**a**) before (0 W) and (**b**) after 10 weeks (10 W) of a simulated degradation test. The 2*θ* positions of hydroxyapatite, brushite, and halite are marked with **△**, **◊**, and **◯**, respectively, followed by the same colour droplines.

XRD patterns of PLA and PLA-HAp composite films before the degradation (0 W) and after 10 weeks (10 W) of the simulated degradation test are shown in Figure 2. An amorphous diffraction pattern of the PLA sample at 0 W, characteristic of a noncrystalline polymer material, is present in all XRD patterns at 0 W and 10 W. The XRD data for composite samples were a good match to the line pattern for crystalline HAp (ICCD 9-432), which increased in intensity at higher hydroxyapatite content.

After 10 weeks of simulated degradation, besides the expected diffraction patterns belonging to amorphous PLA and crystalline HAp, additional crystalline peaks were visible, as seen in Figure 2b. The XRD diffraction pattern of PLA at week 10 showed diffraction peaks at Bragg angles 2*θ* = 31.6, 45.4, and 56.4 attributed to the strongest crystallographic planes (2 0 0), (2 2 0), and (2 2 2) of sodium chloride (halite, NaCl). The presence of sodium and chlorine was confirmed by the elemental composition of the PLA 10 W sample with an energy-dispersive X-ray (EDX) analysis. The characteristic EDX spectra and the acquired elemental surface mapping are presented in Appendix A.

PLA-10-HAp sample at week 10 showed the strongest diffraction peak of brushite at Bragg angles 2*θ* = 11.6. Brushite (CaHPO_4_ × 2 H_2_O) is a phosphate mineral that can appear as an intermediary or final mineral within the active calcium phosphate equilibrium [18,19]. As PBS mainly contains phosphate, sodium, and chloride ions, the formation of both halite and brushite is expected and explained by the dissolution and precipitation processes between the buffered solution and tested samples.

### 3.3. Thermogravimetric Analysis

**Figure 3:**
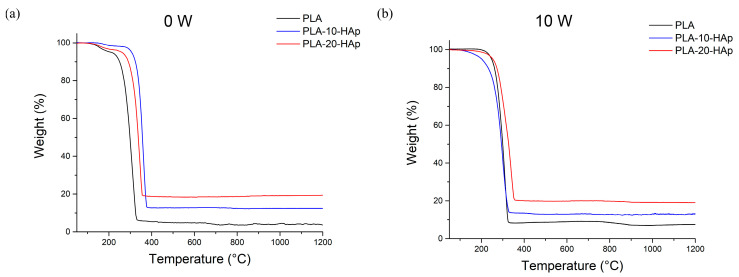
The comparative plot of thermogravimetric analysis curves corresponding to neat PLA and composite samples PLA-10-HAp and PLA-20-HAp (**a**) before (0 W) and (**b**) after 10 weeks (10 W) of simulated biodegradation test.

Thermogravimetric analysis was performed to determine the amount of HAp in the prepared composite films (Figure 3). Weight loss of the samples until 350 °C can be attributed to the single-step PLA thermal degradation. The total weight loss of the neat HAp sample is 5.0 wt.% [20]; thus, the addition of HAp in composites should result in a proportional increase in residual mass.

Thermogravimetric results of samples before in vitro degradation (Figure 3a) show that the remaining weight for the PLA, PLA-10-HAp, and PLA-20-HAp samples are 3.8 wt.%, 12.5 wt.%, and 19.4 wt.%, respectively. Total weight loss follows the assumptions of significant polymer weight loss and residue, which corresponds to the inorganic filler content. Results after 10 weeks of simulated biodegradation in the PBS follow the same behaviour (Figure 3b), although the remaining weight at 1200 °C for the PLA sample doubled due to the presence of an additional thermally stable component. The remaining weight for the PLA, PLA-10-HAp, and PLA-20-HAp after 10 weeks is 7.4 wt.%, 13.0 wt.%, and 19.1 wt.%, respectively. The increase for PLA from 3.8 wt.% at 0 to 7.4 wt.% after 10 weeks is attributed to the NaCl residue. The NaCl presence originated from the PBS agrees with the results of XRD and EDX analysis (Appendix A).

In addition to total weight loss, the thermogravimetric analysis has indicated changes in the thermal stability of prepared samples. Depending on the degree of crystallinity, molecular weight, the PLA enantiomer ratio and polymer purity, the degradation temperature of PLA polymer usually varies between 290–380 °C [21,22]. Each of the mentioned PLA properties can significantly influence thermal and degradation properties and cannot be considered in an isolated way. Because of this, contradictory conclusions on how different parameters impact polymer behaviour can be found in the literature [23,24,25,26]. Different formulations of the polymeric matrix with compounds—such as bioactive glasses, calcium phosphates, starch, and copolymers—made general conclusions almost invalid [27,28,29,30]. Thus, each research path must start with observing basic relations between different parameters towards a particular behaviour path.

As seen in Figure 3a, the thermal degradation temperatures of prepared films before in vitro degradation are 300 °C, 335 °C, and 350 °C for PLA, PLA-10-HAp, and PLA-20-HAp, respectively. Rise in temperature indicates that the addition of HAp improves the thermal stability of the polymer in prepared composites. Similarly, Albano et al. [31] reported that the addition of 30% HAp to the PLLA matrix improves the thermal stability of the polymer. In more detail, HAp increases the polymer activation energy and initial decomposition temperature, but once the degradation process is initiated, the degradation rate is higher. Recently, Tazibt et al. [32] reported that fine dispersion of HAp can increase the degradation temperature in PLA composite, while higher filler content with nonideal dispersion can have the opposite effect on the properties. Compared with the neat PLA, the thermal degradation curves of our composite samples are shifted to higher temperatures, which could indicate a good dispersion of HAp in the matrix.

The onset degradation temperature of the 10 W samples is around 290 °C for PLA and PLA-10-HAp, and 320 °C for PLA-20-HAp. The lower thermal degradation temperatures, compared with the 0 W samples, indicate that samples went through the in vitro degradation process. PLA-20-HAp sample kept the highest thermal degradation temperature and can be considered the most stable one.

Additionally, a slight change in curve shape in the temperature range of 40–300 °C is visible between TGA curves before and after the in vitro degradation (Figure 3a,b). All curves in Figure 3a show the shoulder around 210 °C, which is not present on curves in Figure 3b, and weight loss up to 5% depending on the HAp content. The appearance of the shoulder is not caused by the hydrophilic HAp filler addition, as it appears both on the samples with and without HAp. Detailed studies describing pathways of PLA thermal degradation have been reported [22,33,34]. The most accepted study supposes that the main degradation of poly(lactide) at lower temperatures involves a non-radical backbiting ester interchange reaction, which leads mostly to cyclic oligomers [22]. At higher temperatures, ketones and carbon monoxide are the main degradation products obtained as a result of a radical chain scission mechanism. Albano et al. [31] investigated the degradation behaviour of a similar PLLA-HAp composite. While the main degradation step was around 360 °C, using the derivative graph of thermal degradation, they distinguished a shoulder around 340 °C. In our case, the weight loss obtained at temperatures below 300 °C might have originated from the solvent residue that remained after solvent casting at low temperatures.

As 10 W samples went through 10 weeks of simulated in vitro degradation in PBS, hydrolysis of hydrophobic polymer into more hydrophilic nature oligomers should affect and enhance chemical and physical water binding inside the composite structure. The PLA-10-HAp sample shows a gradual weight loss before the main PLA thermal degradation, unlike the PLA and PLA-20-HAp samples that do not show it.

### 3.4. Glass Transition Temperatures and Sample Disintegration

**Figure 4:**
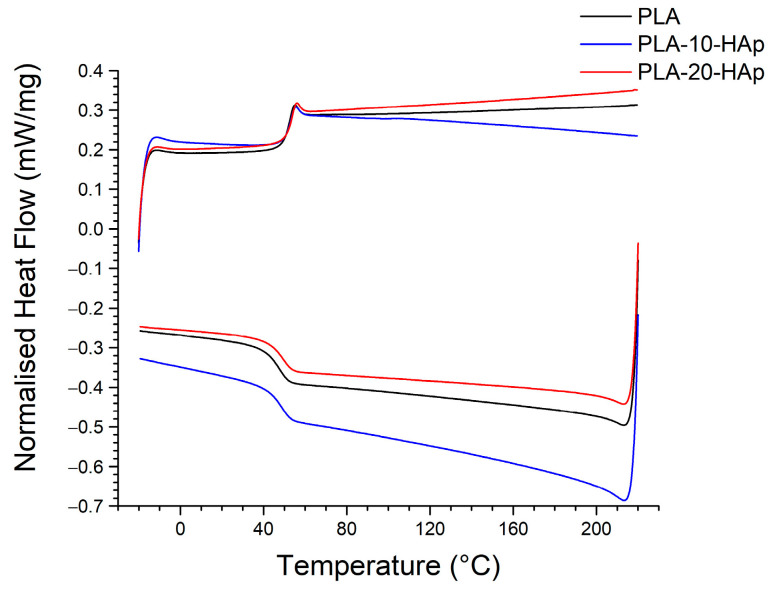
Dynamic differential scanning calorimetry curves for PLA, PLA-10-HAp, and PLA-20-HAp samples. Data were acquired with a temperature program consisting of a heating cycle from −20 °C to 220 °C (upper curves), an isothermal step for 5 min and returning cooling step from 220 °C to −20 °C (lower curves). The heating/cooling rate is 10 °C min^−1^ within an inert nitrogen atmosphere (50 mL min^−1^).

The glass transition temperature (*T*_g_) of PLA usually ranges from 50 °C to 80 °C and is highly dependent on the molecular weight, purity, crystallinity ratio, and thermal history of the polymer [35]. PLA used in this research is specified as a medical-grade PLLA with *M*_w_ ≈ 60 000. Our preliminary analysis of the polymer material showed that it is highly amorphous. The XRD analysis (Figure 2a) and the DSC measurement curves (Figure 4) confirm that there is no crystallisation peak in the PLA sample, even after the addition of HAp. The glass transition temperature of poly(lactic acid) film is 52.4 ± 0.6 °C. The addition of HAp has not affected the glass transition temperature. *T*_g_ remains at 52.7 ± 0.6 °C for PLA-10-HAp and 52.0 ±1.6 °C for PLA-20-HAp, which is considered to be within the experimental error.

As already mentioned in Section 2.2., samples were prepared by solvent casting into concave silicone moulds to obtain films with reproducible shape and mass. Every sample had a thicker middle part of the film (up to 0.5 mm) and a thinner outer part (down to 0.2 mm as it comes to the edge). Under the assumption of uniform hydrolytic degradation, the outer thinner part of a sample should reduce in thickness until its complete disappearance, while the inner thicker part should remain stable.

**Figure 5:**
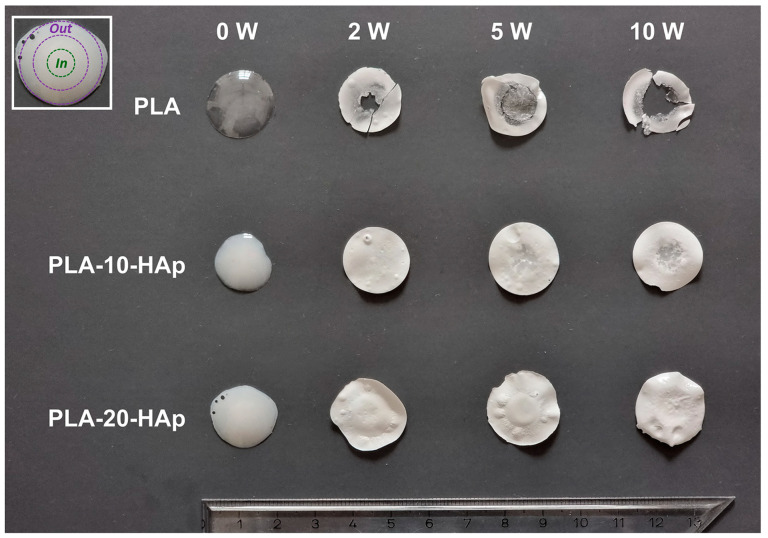
Presentation of the visual sample nonuniformity and disintegration of investigated PLA, PLA-10-HAp, and PLA-20-HAp composites before (0 W) and after 2, 5, and 10 weeks of the in vitro degradation (2 W, 5 W, and 10 W, respectively). The regions defined as the inner (*In*) and outer part (*Out*) of the film are schematically presented in the upper left corner.

After 10 weeks of in vitro hydrolytic degradation, the PLA sample becomes visually nonuniform and disintegrated, as presented in Figure 5. Possible disintegration behaviour was initially indicated by the rapid exponential weight loss in the last weeks of tested biodegradation (Figure 1). The DSC measurement of PLA after 8 weeks showed that the inner thicker part of the film attained a glass transition temperature of 25.8 ± 2.8 °C. The glass transition temperature of the outer part of the same film remained at 44.5 ± 2.5 °C.

The difference in *T*_g_ may indicate that the hydrolytic degradation of PLA is an autocatalytic reaction that is faster at thicker parts of the film. Athanasiou et al. [36] reported that less porous materials tend to hold in the acidic breakdown products, leading to an acceleration of the hydrolytic process. Degradation products locally reduce the pH, which further promotes the polymer degradation process. On the thinner outer part of our prepared films, degradation products are easily washed out, but pH remains stable and the polymer material retains its properties for a longer time.

**Figure 6:**
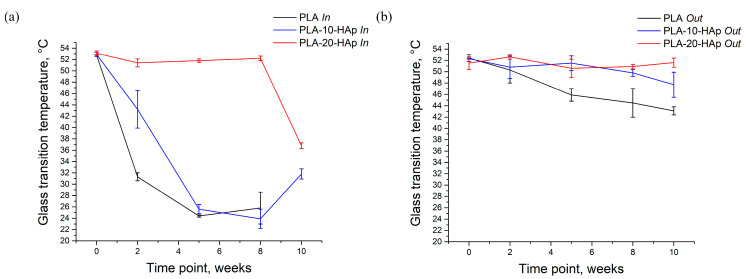
Glass transition temperatures of PLA, PLA-10-HAp, and PLA-20-HAp samples during 10 weeks of degradation in PBS at 37 °C; (**a**) corresponds to the glass transition temperature of the middle circle part of prepared films (*In*). (**b**) presents the *T*_g_ of the outer edge portion of prepared films (*Out*). It was not possible to determine the *T*_g_ of the PLA *In* after 10 weeks of degradation since the middle part of the sample had fully deteriorated.

The disintegration behaviour has led us to determine the glass transition temperatures of the inner (*In*, data presented in Figure 6a) and outer (*Out*, data presented in Figure 6b) parts of all samples. Summarized glass transition temperature results are presented as data tables in Appendix B. From Figure 6a it is obvious that as time increases, the *T*_g_ shifts to lower temperatures. During the hydrolysis process, the scission of the longer polymer chains occurs and leads to a decrease in PLA molecular weight, which results in a decrease in the glass transition temperature.

The *T*_g_ of the inner part of the PLA sample decreased to 31.3 ± 0.7 °C and 24.4 ± 0.2 °C after 2 and 5 weeks, respectively. As the inner part of the sample degraded significantly, *T*_g_ measured for PLA after 8 weeks increased to 25.8 ± 2.8 °C. It can be assumed that the degradation products act as plasticizers for polymer molecules and that with the progress of the degradation, they come out of the sample more easily. This may be the reason why the sample after 8 weeks of degradation (with less “plasticizer”) shows a higher *T*_g_ compared with the *T*_g_ of the sample after 5 weeks of degradation. Due to the sample degradation, it was impossible to collect the inner part of PLA 10 W for DSC analysis.

The same behaviour of the inner part during degradation is observed in the PLA-10-HAp sample with a slight time delay (Figure 6a). Significant visual degradation and overall sample weight loss of composite samples did not occur within the 10-week degradation period, but *T*_g_ measurements confirmed that the inner part of the sample degraded significantly faster than the outer part. In comparison with PLA 10 W sample, PLA-10-HAp 10 W did not show any significant weight loss, which could indicate composite degradation. The presence of bioresorbable HAp allows for a continuous process of dissolution and precipitation, which, in the end, affects the weight of the degraded composite film. Pitt et al. [37] and Li et al. [38] showed that the decrease in molecular weight, accompanied by a *T*_g_ decrease, occurs much earlier than the material weight loss is observed [37,38]. Our in vitro degradation results agree with these findings.

The PLA-20-HAp sample visually remained almost uniform, which could indicate that polymer degradation had not yet occurred. The DSC analysis confirmed that until 8 weeks of in vitro degradation, the *T*_g_ of the inner and outer parts of the PLA-20-HAp sample remained constant, at around 52 °C. After 10 weeks of degradation, PLA-20-HAp inner part showed a significant decrease in *T*_g_. Still, we can assume that a greater amount of HAp is responsible for the slower PLA degradation.

*T*_g_ of the PLA sample decreased from the initial 52.4 ± 0.6 °C below the physiological temperature of 37 °C after a few weeks of the simulated in vitro degradation. It is straightforward that its degradation properties significantly affect the mechanical stability of a potential implant and should be carefully modified for biomedical applications.

The analysis of DSC data (Figure 6) combined with the weight loss results (Figure 3) leads us to the unambiguous conclusion that a greater amount of hydroxyapatite in medical-grade PLA/HAp composite films slows down the degradation process.

These results are, at least partially, in disagreement with many other studies where the degradation of different PLA-HAp composites was enhanced as HAp content was increased [39,40,41]. Zhang et al. [10] recently reported a comprehensive study in which the quality of the 3D-printed scaffold decreased significantly as the degradation time increased. It is assumed that highly hydrophilic HAp particles help water infiltration, which should result in faster degradation, and that the degradation of PLA is mainly related to the solvent environment and acidic products from a scaffold or in vivo response [42]. Xu et al. [43] studied grafted PLA-HAp composite fibres and hypothesised that low HAp content may delay degradation due to the reduction of autocatalytic degradation, but that enhanced wettability, with higher quantities of HAp, is the reason for the increased degradation rate.

Alex et al. [44] studied the relative role of polymer chain scission and solvation in the reduction of mechanical properties of degrading PLA and concluded that solvation plays a more active role. Based on our in vitro degradation results, it might be assumed that the hydrophilic nature of the HAp nanoparticles could disrupt the solvation, i.e., there is less water available to interact with the polymer.

In either case, further research with a 3D-structured composite material and dynamic in vitro conditions should bring new information about the behaviour of our medical-grade PLA/HAp composite.

### 3.5. Sample Morphology

**Figure 7:**
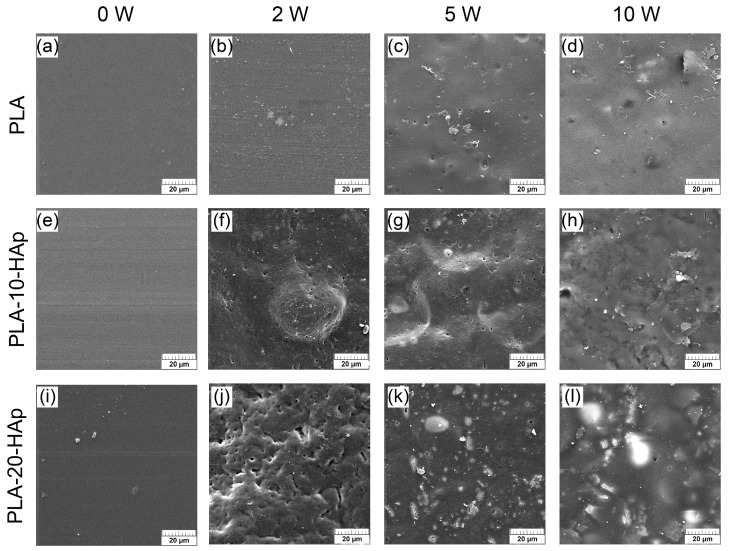
SEM micrographs of samples during 10 weeks of degradation in PBS at 37 °C. Micrographs (**a**–**d**) present the surface change of PLA film. The degradation behaviour of PLA-10-HAp composite film is shown on micrographs (**e**–**h**). Micrographs (**i**–**l**) show surface changes of PLA-20-HAp composite film. Representative images are shown at 2000 × magnification and the scale bars represent 20 µm.

The degradation of the neat PLA film and the composite PLA/HAp films in PBS at 37 °C was characterised by SEM as well. All micrographs before degradation (0 W) show a smooth surface, with few aggregated HAp particles on the surface of the PLA-20-HAp film (Figure 7i). After 2 weeks of degradation, the PLA surface (Figure 7b) indicates the appearance of scattered microparticles, whose size and presence become more significant as degradation is prolongated (Figure 7c,d). Observed microparticles are assumed to be halite crystals precipitated from the PBS, identified by the XRD, TGA, and EDX analysis (Section 3.2 and Section 3.3 and Appendix A).

The surface of composite films shows noticeable changes with the degradation time. The smooth surface of initial samples (Figure 7e,i) turns into a pitted structure after 2 weeks of degradation (Figure 7f,j), where the higher amount of HAp changes hole dimensions from a submicron size to a micrometre range. At 5 W surface becomes more closed and the aforementioned holes are filled with brighter material. After 10 weeks of degradation, PLA-20-HAp surface (Figure 7l) is composed of a significant amount of white regions than the PLA-10-HAp film surface (Figure 7h). An energy-dispersive X-ray (EDX) analysis of the PLA-20-HAp surface, presented in Appendix C, shows that brighter areas match the distribution of calcium and phosphorus.

**Figure 8:**
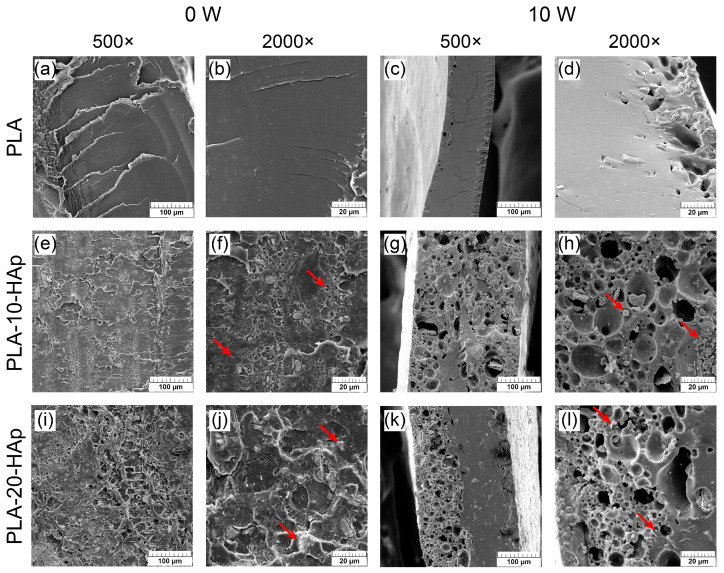
SEM micrographs of the cross-sectional surface before degradation (0 W) and after a 10-week degradation period in PBS at 37 °C (10 W). Micrographs (**a**–**d**) present cross-sections of PLA film, (**e**–**h**) are cross-sections of PLA-10-HAp and (**i**–**l**) show cross-sections of PLA-20-HAp composite film. 0 W cross-sections represent the inner part of the films, while 10 W micrographs represents cross-sections of the thinner outer part of samples as the inner part of PLA film disintegrated during degradation. Representative images are shown at 500× and 2000× magnification with the scale bars that represent 100 and 20 µm. Red arrows indicate calcium-phosphate agglomerates within PLA matrix.

In addition to the surface analysis of the films, samples’ cross-sections are analysed by SEM as well. Figure 8 presents cross-sections of samples at two different magnifications (500× and 2000×) before and after degradation (0 W and 10 W).

Micrographs in Figure 8 at both magnifications before degradation (0 W) reveal that HAp is relatively evenly distributed through the whole cross-section of the inner sample part. The biggest agglomerates in PLA-10-HAp and PLA-20-HAp samples remained under 5 and 10 µm, respectively, while most of the particles were successfully blended within the polymer matrix. In spite of the hydrophobic nature of PLA, hydrophilic HAp improved the thermal properties and slowed down the degradation of PLA.

The cross-section of the outer part of the PLA film (Figure 8c) became very thin (approx. 0.1 mm), which agrees with the significant weight loss in the last weeks of degradation. Holes are dominant on one side of the PLA film, whereas the larger portion of the remaining film seems nonporous on the micrometric level. Cross-section micrographs of composites after 10 W degradation (Figure 8g,h,k,l) reveal that degradation occurs from the inside of composite samples. As active dissolution/precipitation of inorganic components (HAp, brushite, halite) pitted the sample surface, it caused a similar effect on the film interior. Cross-sections of PLA-10-HAp and PLA-20-HAp show micrometric pores that form a nonhomogeneous interconnected structure. It could be assumed that due to the degradation of PLA, there is also a redistribution of HAp particles within the matrix. It seems that particles settled down, and this effect is more obvious in composite films with a higher HAp content. This is especially noticeable with the sample PLA-20-HAp.

It would be interesting to study how such significant changes in the composite structure during degradation, from bulky to porous, affect its mechanical properties. This issue will be covered in our upcoming research.

## 4. Conclusions

Composite films based on medical-grade PLA and hydroxyapatite synthesized from a biogenic source were prepared by solvent casting technique. The addition of relatively evenly distributed inorganic bioactive filler in the PLA matrix improved the thermal stability of the prepared composites.

The degradation behaviour of prepared composite films incubated in phosphate-buffered saline solution at 37 °C was examined during a 10-week period. The hydrolytic degradation of PLA is an autocatalytic reaction that is faster at thicker parts of the film. The inner thicker part of the material, with an initial *T*_g_~50 °C, degraded to the state where the glass transition temperature was below the physiological temperature. The decrease in glass transition temperature was seen before the weight loss of composite samples. The degradation caused PLA sample nonuniformity and disintegration in the last weeks of degradation. Slowed autocatalytic degradation in composite samples is indicated by the more stable glass transition temperatures compared with the neat PLA samples. SEM analysis of cross-sections of composite films showed noticeable changes with degradation time.

We can assume that a greater amount of HAp is responsible for the slower degradation of medical-grade PLA. It should be emphasized that great attention should be directed towards comparing the results of technical- and medical-grade PLA.

## Figures and Tables

**Figure A1:**
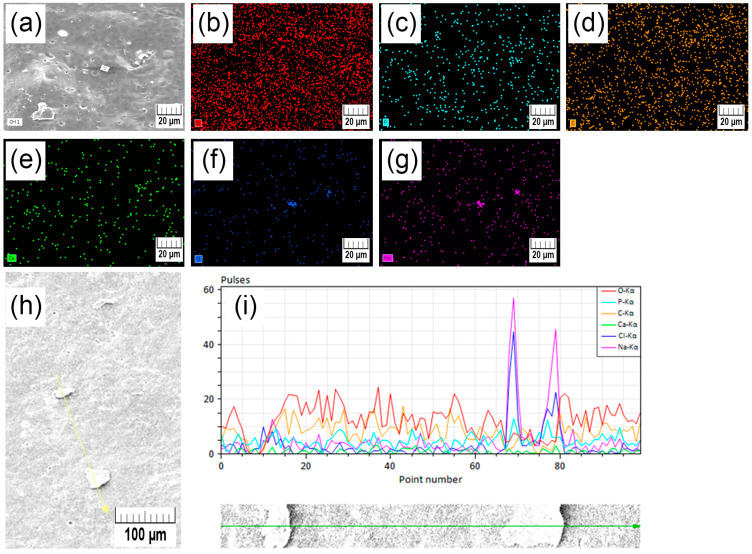
An energy-dispersive X-ray (EDX) analysis of the PLA surface after 10 weeks of in vitro degradation (**a**). Figures represent the distribution of oxygen (**b**), phosphorous (**c**), carbon (**d**), calcium (**e**), chlorine (**f**), and sodium atoms (**g**) on the same surface area. Scale bars represent 20 µm. At the bottom of the figure, a line scan analysis trace is shown (**h**) where scale bars represent 100 µm. Line scan analysis resulted in spectra where peak values correspond to the frequency of detected atoms on a traced path (**i**).

**Figure A2:**
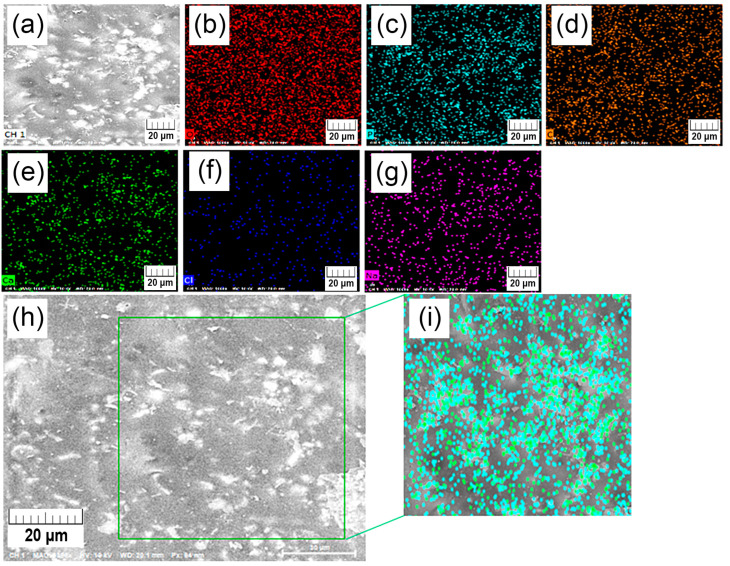
An energy-dispersive X-ray (EDX) analysis of the PLA-20-HAp surface after 10 weeks of in vitro degradation (**a**). Figures represent the distribution of oxygen (**b**), phosphorous (**c**), carbon (**d**), calcium (**e**), chlorine (**f**), and sodium atoms (**g**) on the same surface area. In the bottom detailed topography of surface (**h**) the topography of phosphorus (turquoise) and calcium (green) atoms is shown separately (**i**). Scale bars represent 20 µm.

## Data Availability

The data presented in this study are available on request from the corresponding author.

## Appendix A

An energy-dispersive X-ray analysis by the elemental composition mapping (Figure A1a–g) and line scan analysis (Figure A1h,i) confirmed the presence of sodium and chlorine atoms in the PLA sample after 10 weeks of in vitro degradation. NaCl precipitates in crystalline-like agglomerates with a size of a few micrometres. The appearance of phosphorous (Figure A1c) and calcium atoms (Figure A1e) is confirmed and originates from the calcium-phosphate precipitates from PSB.

## Appendix B

The glass transition temperatures of the inner (*In*, data presented in Table A1) and outer (*Out*, data presented in Table A2) parts of PLA, PLA-10-HAp and PLA-20-HAp samples during the in vitro degradation.

**Table A1:** Glass transition temperatures of the middle circle part, *T*_g_ (*In*), of PLA. PLA-10-HAp and PLA-20-HAp films during 10 weeks of degradation in PBS at 37 °C.

	*T*_g_ (*In*)*/*°C					
Degradation Week	PLA		PLA-10-HAp		PLA-20-HAp	
0	52.8	±0.1	52.9	±0.4	53.1	±0.4
2	31.3	±0.7	43.2	±3.3	51.4	±0.7
5	24.4	±0.2	25.6	±0.8	51.8	±0.3
8	25.8	±2.8	23.9	±1.7	52.2	±0.4
10	- ^1^	-	31.8	±0.9	36.8	±0.5

^1^ inner part of the PLA after 10 weeks deteriorated fully.

**Table A2:** Glass transition temperatures of the outer edge portion, *T*_g_ (*Out*), of PLA. PLA-10-HAp and PLA-20-HAp films during 10 week of degradation in PBS at 37 °C.

	*T*_g_*(Out)/*°C					
Degradation Week	PLA		PLA-10-HAp		PLA-20-HAp	
0	52.4	±0.6	52.3	±0.1	51.5	±1.1
2	50.3	±2.3	50.8	±2.0	52.6	±0.4
5	45.9	±1.1	51.5	±1.3	50.6	±1.6
8	44.5	±2.5	49.8	±0.6	50.9	±0.4
10	43.1	±0.7	47.7	±2.2	51.6	±0.8

## Appendix C

Results of an energy-dispersive X-ray analysis of the PLA-20-HAp surface after 10 weeks of in vitro degradation are shown in Figure A2. The appearance of calcium and phosphorous origins from HAp, oxygen, and carbon atoms from PLA, while chlorine and sodium originate from halite precipitates from PBS. The distribution of the white areas on the composite surface (Figure A2a) overlaps with the most intensive areas of phosphorous (Figure A2c) and calcium atoms (Figure A2e). A detailed view of the sample surface (Figure A2h) shows that the brighter white regions correspond to the most intensive areas of phosphorous (turquoise) and calcium (green) atoms (Figure A2i).
